# Interactions between metabolism and chromatin in plant models

**DOI:** 10.1016/j.molmet.2020.01.015

**Published:** 2020-02-12

**Authors:** Christian Lindermayr, Eva Esther Rudolf, Jörg Durner, Martin Groth

**Affiliations:** Institute of Biochemical Plant Pathology, Helmholtz Zentrum München, German Research Center for Environmental Health, Ingolstädter Landstrasse 1, 85764 München/Neuherberg, Germany

**Keywords:** Nitric oxide, Reactive oxygen species, Redox modification, Methionine cycle, Folate metabolism, Chromatin, Histone modification, DNA methylation, Plants

## Abstract

**Background:**

One of the fascinating aspects of epigenetic regulation is that it provides means to rapidly adapt to environmental change. This is particularly relevant in the plant kingdom, where most species are sessile and exposed to increasing habitat fluctuations due to global warming. Although the inheritance of epigenetically controlled traits acquired through environmental impact is a matter of debate, it is well documented that environmental cues lead to epigenetic changes, including chromatin modifications, that affect cell differentiation or are associated with plant acclimation and defense priming. Still, in most cases, the mechanisms involved are poorly understood. An emerging topic that promises to reveal new insights is the interaction between epigenetics and metabolism.

**Scope of review:**

This study reviews the links between metabolism and chromatin modification, in particular histone acetylation, histone methylation, and DNA methylation, in plants and compares them to examples from the mammalian field, where the relationship to human diseases has already generated a larger body of literature. This study particularly focuses on the role of reactive oxygen species (ROS) and nitric oxide (NO) in modulating metabolic pathways and gene activities that are involved in these chromatin modifications. As ROS and NO are hallmarks of stress responses, we predict that they are also pivotal in mediating chromatin dynamics during environmental responses.

**Major conclusions:**

Due to conservation of chromatin-modifying mechanisms, mammals and plants share a common dependence on metabolic intermediates that serve as cofactors for chromatin modifications. In addition, plant-specific non-CG methylation pathways are particularly sensitive to changes in folate-mediated one-carbon metabolism. Finally, reactive oxygen and nitrogen species may fine-tune epigenetic processes and include similar signaling mechanisms involved in environmental stress responses in plants as well as animals.

## Highlights

• Plant specific non-CG methylation pathways are particularly sensitive to changes in folate-mediated one-carbon metabolism.
• ROS and NO coordinate metabolic pathways and gene activities involved in chromatin modulation.
• ROS and NO might also be pivotal in mediating chromatin dynamics during environmental stress responses.
• Mammals and plants share a common dependence on metabolic intermediates that serve as cofactors for chromatin modulation.

## 1 Introduction

DNA is packaged inside eukaryotic nuclei by wrapping around histone proteins. A total of 147 base pairs are fitted around each histone octamer consisting of two copies of each core histone H2A, H2B, H3, and H4. The resulting nucleosomes together with associated non-histone proteins and RNA comprise the chromatin. Histones can be modified by post-translational attachment or removal of small chemical groups such as acetyl and methyl. In addition, bases of DNA molecules are also subject to chemical modification, most prominently cytosine methylation at position 5 of the pyrimidine ring (5-methylcytosine). DNA methylation and post-translational histone modifications have important functions in gene regulation by influencing the chromatin structure and accessibility of regulatory DNA elements. Transmitted during cell division, chromatin modifications can lead to persistent morphological and physiological changes and are therefore considered epigenetic marks. The enzymes that catalyze these modifications depend on metabolic intermediates as cosubstrates or cofactors. Although this link between metabolism and epigenetic regulation is evident, the appreciation of the biological implications and underlying mechanisms has just begun. Seminal studies have demonstrated the importance of metabolic regulation in mammalian reprogramming [1,2]. It is now becoming apparent that metabolism not only serves as a chemical supplier for chromatin modifications, but that the interaction of central metabolic pathways and epigenetic regulation is decisive for cell fate during development and disease [3].

The metabolism of plants is complex and highly adaptive to environmental changes. Specialized pathways produce “secondary” metabolites that allow plants to tolerate adverse abiotic conditions, defend themselves against pathogens and herbivores, and communicate with their surroundings [4]. Secondary metabolic pathways can impose high demands for intermediates from primary metabolism. Consequently, primary metabolic pathways must be able to respond dynamically while sustaining central cellular functions [5]. Reactive oxygen species, nitric oxide, and glutathione are particularly important for plant environmental responses, as they fulfill integral stress signaling functions and regulate cellular redox homeostasis [6]. Moreover, photosynthesis implies plant-specific pathways and diurnal metabolic patterns, for example, in connection to starch metabolism [7]. As these pathways converge on central intermediates, including S-adenosyl methionine, α-ketoglutarate, and acetyl-CoA, which are known to be required for chromatin modifications, it is likely that plants have evolved epigenetic mechanisms that integrate metabolic dynamics during development and environmental responses.

Compared to animal models, the territory of plant “metaboloepigenetics” is largely uncharted. To date, most insights are based on forward genetic screens aimed at the identification of gene silencing mechanisms and revealing many RNA and chromatin-associated factors, but also components of metabolic pathways involved in cofactor regulation [[8], [9], [10], [11], [12]]. Approaches that specifically address the connections between metabolism and epigenetics in plants remain sparse. Therefore, this review provides a comparison of current insights in the mammalian and plant fields. Due to their prevalence and for the sake of space, we will focus on chromatin acetylation and methylation and the metabolism of the involved cofactors. Finally, we will put the described links between metabolism and chromatin dynamics in a biological context to discuss potential causes of environmentally induced epigenetic changes and how these changes might contribute to stress resistance in plants.

## 2 Histone acetylation and acetyl-CoA metabolism

### 2.1 Overview of histone acetylation and deacetylation in plants

Post-translational modifications (PTMs) of histone proteins include acetylation, methylation, sumoylation, ubiquitinylation, phosphorylation, glycosylation, and carbonylation of different histone residues. The addition and removal of these modifications by chromatin-modifying complexes makes the chromatin structure very dynamic and allows fine-tuning of the accessibility of the DNA to transcription factors. Because of the important function of histone acetylation on chromatin structure, this modification has been intensively investigated for several decades. Acetylation of histones typically occurs at the N-terminal tails, which are rich in lysine residues, and neutralizes the positive charges of the histone tails resulting in a decrease in their affinity for negatively charged DNA. Thus, acetylation of histones is associated with a loosened chromatin structure and often occurs in the promoter and 5′-end of genes enabling transcription factor binding to DNA [13]. Moreover, acetylated histone lysine residues can be recognized by transcriptional co-regulators and chromatin remodeling factors containing a “bromodomain” [14].

The level of histone acetylation is determined by the activity of both histone acetyl transferases (HATs) and histone deacetylases (HDACs). HATs use acetyl-CoA as a cofactor to transfer acetyl groups on histone lysines. They are classified into four distinct families harboring different substrate specificities [[15], [16], [17]]. For instance, HAG1 from the GCN5-like family almost exclusively acetylates H3K14, whereas HAG2 from the same family shows specificity for H4K12. HAM1 and HAM2 belonging to the Myst-like family of HATs both acetylate H4K5, whereas HAC1, 5, and 12 from the CBP-like family have multiple target lysines on H3 and H4. The fourth group called TAF_II_250-like has not been characterized in plants. Some HATs contain bromodomains, enabling them to bind acetylated histones, indicating that acetylation of histones by one HAT may help to recruit other HATs on the same nucleosome [15].

The HDACs in *Arabidopsis thaliana* (herein called *Arabidopsis*) are encoded by 18 genes and can be grouped into three types, silent information regulator 2 (SIR2), reduced potassium dependency 3 (RDP3), and plant-specific histone deacetylase 2 (HD2) [16,17]. The structurally diverse family of sirtuins (2 members in *Arabidopsis*) contains homologs to yeast SIR2. These enzymes catalyze protein deacetylation using NAD^+^ as a cofactor. The RPD3 family consists of 12 members and is characterized by a highly conserved HDAC domain with a catalytic zinc ion [[15], [16], [17]]. The HD2-like family (HD2A, HD2B, HD2C, and HD2D) is plant specific, since no homologs have been identified in other organisms to date [17]. Although HD2-ko plants have altered histone acetylation levels, their HDAC activity is still questioned. It is possible that they are important interaction partners of RPD3-like HDACs required for their activity.

Analysis of mutants affected in either HAT or HDAC revealed that histone acetylation plays key roles in several physiological processes, including cell cycle, flowering time, response to environmental conditions such as light or pathogen attack, root and shoot development, and hormone signaling [16]. These functions of HATs and HDACs are not only associated with chromatin modification, because HATs and HDACs have also been found to be regulate the acetylation of numerous non-histone proteins.

### 2.2 Acetyl-CoA metabolism affects histone acetylation in plants

Acetyl-CoA is a central metabolite involved in numerous anabolic and catabolic pathways and is a substrate for protein acetylation. In mammalian cells, the nuclear and cytosolic acetyl-CoA is supplied by at least three pathways: citrate cleavage, pyruvate dehydration, and acetylcarnitine conversion [[18], [19], [20]]. In the cytosol and nucleus, adenosine triphosphate (ATP)-citrate lyase (ACL) cleaves citrate exported from mitochondria to regenerate acetyl-CoA that can be used for other biosynthetic processes such as fatty acid synthesis and histone acetylation [21]. Moreover, a pyruvate dehydrogenase complex can be translocated from mitochondria to nuclei to generate acetyl-CoA and mediate histone acetylation in mammalian cells under certain conditions [20,22].

In plant cells, the plastids, mitochondria, peroxisomes, and cytosol produce acetyl-CoA [23]. In *Arabidopsis*, it has been shown that cytosolic acetyl-CoA is mainly produced by the ATP-citrate lyase and that reduction in ACL activity leads to complex bonsai phenotypes [23,24]. Cytosolic acetyl-CoA can either be converted to malonyl-CoA by acetyl-CoA carboxylase (ACC) to synthesize very-long-chain fatty acids, flavonoids, and malonyl derivatives, or condensed to acetoacetyl-CoA to synthesize precursors of isoprenoids [25,26]. Two different isoforms of ACC have been described to date. Heteromeric ACC is composed of four subunits and is located in the plastids where it is responsible for *de novo* fatty acid synthesis. Homomeric ACC is composed of a single large polypeptide containing ACC1 or ACC2 and is located in the cytosol or plastids, respectively. It is required for elongating plastid-produced fatty acids. Blocking the function of cytosolic ACC1 leads to elevated levels of acetyl-CoA and consequently to global histone hyperacetylation, predominantly at lysine 27 of histone H3 (H3K27), as shown in *Arabidopsis* [27]. The increase in H3K27ac in *acc1* mutants depends on ACL and the histone acetyltransferase GCN5. Consequences of increased acetyl-CoA levels and H3K27ac are changes in gene transcription and metabolite levels. Gene ontology analysis indicates that H3K27 hyperacetylated genes are enriched in primary metabolic processes including amino acid biosynthesis. The expression of 22 amino acid biosynthesis genes consistently leads to an at least twofold increase in *acc1* [27]. Furthermore, two of the tricarboxylic acid (TCA) cycle intermediates (isocitrate and α-ketoglutarate) significantly over-accumulate in *acc1*. Isocitrate is metabolized to α-ketoglutarate by isocitrate dehydrogenase. Interestingly, α-ketoglutarate is an important metabolic intermediate that acts as a cofactor for several chromatin-modifying enzymes, including histone demethylases [28].

Another study revealed a link between peroxisomal acyl-CoA oxidase 4 (ACX4), an enzyme in the fatty acid *β*-oxidation pathway, histone acetylation, and DNA methylation-mediated gene silencing [29]. Screening for suppressor of *pro35S:NPTII* silencing, the authors identified an *acx4* mutant with reduced overall levels of H3Ac and H4Ac and increased DNA methylation at some endogenous genomic loci, which resulted in enhanced transcriptional silencing of reporter and some endogenous genes [29]. Thus, ACX4 activity of the fatty acid *β*-oxidation in peroxisomes is closely linked to nuclear epigenetic modifications, which may affect diverse cellular processes in plants. In sum, fluctuations in acetyl-CoA levels in the cytosol or organelles affect histone acetylation in eukaryotic cells [24,27,30].

## 3 Chromatin methylation and one-carbon metabolism

### 3.1 DNA and histone methylation pathways in plants

In addition to histone acetylation, DNA and histone methylation are among the most studied and conserved chromatin modifications. Although DNA methylation can be lost in some eukaryotes, its common role is the silencing of transposable elements. Moreover, DNA methylation is associated with additional functions that involve long-term gene inactivation such as imprinting and X-inactivation. Loss of DNA methylation is lethal to mammalian embryos and often leads to severe developmental defects in plants, but is tolerated in *Arabidopsis*, which makes it a very useful model for genetic dissection of the involved pathways. In contrast to mammals, where DNA methylation is almost exclusively found at CG sites, most plants also contain CHG and CHH (H = C, A, or T) methylation [31]. In *Arabidopsis*, DNA methylation in all sequence contexts is established by the RNA-directed DNA methylation (RdDM) pathway, which includes the plant-specific Pol II-related RNA polymerases Pol IV and V and components of RNA interference [31]. Once established, symmetric CG methylation (that is, CG sites that are methylated on both strands) is propagated during replication by the recruitment of maintenance DNA methyltransferase MET1 (a homolog of mammalian DNMT1) to hemimethylated DNA at the replication fork [31]. This mechanism is conserved between mammals and plants. CHH methylation is either maintained by RdDM, mostly acting on the euchromatic chromosome arms, or by plant-specific DNA methyltransferase CMT2, whereas CHG methylation is maintained by its paralog CMT3 [31]. Cross-talk between and self-reinforcing loops within the DNA methylation pathways ensure the maintenance of non-CG methylation patterns. The feedback mechanism involved is illustrated by the interplay of non-CG methylation and di-methylation of lysine 9 on histone H3 (H3K9me2), which is catalyzed by the SET domain-containing histone methyltransferases (HMTs) KYP/SUVH4, SUVH5, and SUVH6: CMT2 and CMT3 bind nucleosomes containing H3K9me2 deposited by the HMTs, which in turn bind to cytosines methylated by CMT2 and CMT3 [32]. This mechanism leads to the genome-wide association of non-CG and H3K9 methylation [33]. Moreover, mammalian genomes undergo global erasure and re-establishment of most DNA methylation marks in pre-implantation development and during gametogenesis. In contrast, global resetting of DNA methylation in *Arabidopsis* does not seem to occur in generative nuclei of the gametes or during embryogenesis [34]. Accordingly, DNA methylation patterns are generally stable and transgenerational inheritance of epialleles is common in *Arabidopsis* and other plants [34].

Histone methylation marks are commonly found on arginine and lysine residues of the N-terminal tails of histones H3 and H4 (for the sake of space, we will hereinafter only consider lysine methylation). Lysine methylation in general depends on methyltransferases with a catalytic SET domain and surrounding conserved amino acid sequences that together specify the target and number of methyl groups added (up to 3) [35]. Depending on the position of the lysine residue and degree of methylation, these marks are associated with different transcriptional states [36]. For example, highly conserved H3K4me3 is enriched in promoters of active genes, whereas H3K27me3 marks inactive genes that are controlled by the Polycomb Group proteins and often have important developmental functions in animals as well as plants [37,38].

DNA and histone methylation are reversible and active reversal plays important roles during development and environmental responses. As previously mentioned, animal and plant genomes display fundamental differences in their DNA methylation dynamics, which are also reflected in their DNA demethylation mechanisms. Animals rely on ten-eleven translocation (TET) proteins that can stepwise oxidize 5-methylcytosine to 5-hydroxymethylcytosine, 5-formylcytosine, and 5-carboxylcytosine [[39], [40], [41]]. Once oxidized, replication leads to “passive” replacement of 5-methylcytosine by cytosine. Alternatively, 5-formylcytosine and 5-carboxylcytosine are actively excised and replaced by cytosine through thymine DNA glycosylase and base excision repair [42]. In contrast, plants lack genes that encode TET proteins [39]. Instead, active DNA demethylation is achieved through the excision of 5-methylcytosine by a family of DNA glycosylases, including DME, DML2 and 3, and ROS1, followed by cytosine replacement through the base excision repair pathway [43]. DME activity in the central cell ensures genomic imprinting in the endosperm and is essential for seed development [44]. The main function of ROS1 appears to be the confinement of DNA methylation to its target region to avoid DNA methylation spreading and silencing of adjacent genes [45].

Histone demethylases belong either to the class of flavin adenine dinucleotide (FAD)-dependent amine oxidases or the Fe(II) and α-ketoglutarate-dependent hydroxylases [46]. Members of the first class include LSD1/KDM1 homologs, which play critical roles in cell differentiation and use mono- and di-methylated H3K4 and H3K9 as substrates. Members of the second class are characterized by the Jumonji (Jmj) domain that binds the cofactors Fe(II) and α-ketoglutarate. They form several families that are distinguished by domain architecture and substrate specificity [46]. In *Arabidopsis*, approximately half of the 21 Jmj-coding genes and all four LSD1 homologs have been characterized to date. The described biological functions, frequently fulfilled by partially redundant paralogs, range from facilitating DNA methylation and transposon silencing to negative regulation of flowering and circadian clock genes [[47], [48], [49], [50]].

### 3.2 Changes in the methionine cycle affect transcriptional gene silencing in plants

**Figure 1:**
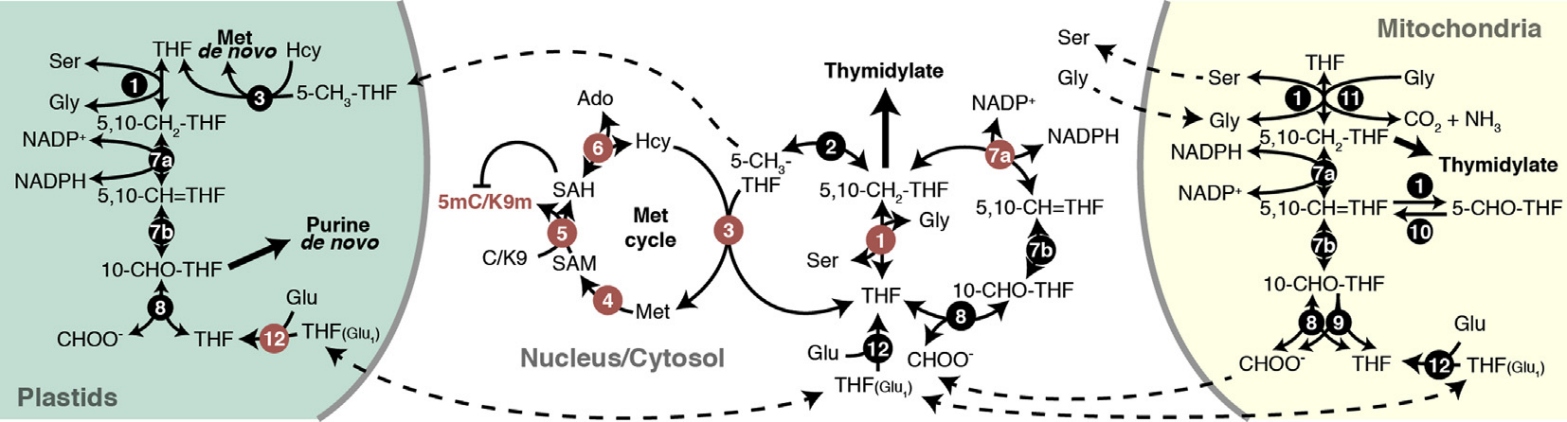
**Folate-mediated C1 metabolism in *Arabidopsis* and its involvement in DNA and histone methylation**. Enzymes with identified mutations that affect DNA and histone H3 lysine 9 methylation (5mC/K9m) in *Arabidopsis* are highlighted in red (further description and citations in main text). (1) Serine hydroxymethyltransferase (SHMT), (2) methylenetetrahydrofolate reductase (MTHFR), (3) methionine synthase (MS), (4) *S*-adenosylmethionine synthetase/methionine adenosyltransferase (SAMS/MAT), (5) DNA/histone methyltransferases, (6) *S*-adenosylhomocysteine hydrolase (SAHH), (7a) methylenetetrahydrofolate dehydrogenase, (7b) methenyltetrahydrofolate cyclohydrolase (7a&7b: MTHFD), (8) 10-formyltetrahydrofolate synthetase (10-FTHFS), (9) 10-formyltetrahydrofolate deformylase (10-FDF), (10) 5-formyltetrahydrofolate cycloligase (5-FCL), (11) glycine decarboxylase complex (GDC), (12) folylpolyglutamate synthetase (FPGS), THF(Glu_1_) and THF refer to mono- and polyglutamylated tetrahydrofolate, respectively.

*S*-adenosylmethionine (SAM) serves as methyl donor for most enzymatic methylation reactions, including cytosine methylation and lysine methylation by DNA and histone methyltransferases, respectively (Figure 1). Accordingly, there is wide evidence, ranging from *Drosophila*, *Caenorhabditis elegans*, and mammalian models to rice and *Arabidopsis*, that deficiencies in SAM production lead to reduced chromatin methylation [11,[51], [52], [53]]. SAM is the central constituent of the methionine cycle and produced by *S*-adenosylmethionine synthetase (SAMS) from methionine (Met) and adenosine triphosphate (ATP) as substrates. Among the four SAMS isoforms in *Arabidopsis*, AtSAMS4 seems to be particularly required for DNA and histone methylation [12]. As reported by the authors, mutations in AtSAMS4 led to decreased levels of SAM, globally reduced non-CG methylation and H3K9me2, and, accordingly, activation of many TEs. Other SAMS isoforms, in contrast, were dispensable for TE silencing, which might be due to functional redundancy or reduced and tissue-specific expression [12].

Upon methyl transfer, SAM is converted to *S*-adenosylhomocysteine (SAH), which *per se* functions as a competitive inhibitor of SAM-dependent reactions due to higher affinities of most methyltransferases toward SAH than toward SAM [ 54]. Accordingly, tissue or plasma SAM/SAH ratios (also known as the methylation index) are often correlated with methylation levels, particularly DNA methylation, and can therefore serve as indicators of the methylation status [56,55]. SAH is reversibly converted to adenosine (Ado) and homocysteine (Hcy) by the enzyme SAH hydrolase (SAHH) [57]. As this constitutes the only way to remove SAH, blocking SAHH activity leads to increased SAH levels, which are often accompanied by feedback accumulation of SAM and Met, but overall lead to decreased SAM/SAH ratios. In plants, DNA methylation and H3K9me2 seem to be particularly sensitive to impaired SAHH activity, as *Arabidopsis sahh1* mutants have been identified multiple times in different screens for transcriptional gene silencing factors [8,[58], [59], [60]]. Moreover, expression of antisense RNA of *SAHH* in tobacco plants resulted in a loss of DNA methylation in repetitive elements [61]. Other studies employed a selective reversible inhibitor of SAHH, dihydroxypropyladenine, in tobacco callus and TBY-2 cells, leading to accumulation of SAH and concomitant DNA hypomethylation [62,63] as well as pleiotropic developmental defects such as decreased apical dominance, altered leaf and flower symmetry, and reduced fertility in treated tobacco plants [64]. *Arabidopsis* contains two SAHH isoforms encoded by *SAHH1* and *SAHH2.* SAHH1 appears to be the main isoform as only *SAHH1* is an essential gene and is generally expressed at higher levels than *SAHH2* [65]. Characteristically, *sahh1* knock-down or hypomorphic mutants display de-repression of many transposable elements (TEs) due to global reduction in H3K9me2 and DNA methylation, non-CG methylation being mostly affected. This is probably due to the simultaneous inhibition of the respective HMTs (KYP/SUV4, SUVH5, and SUVH6) and DNMTs (CMT2 and 3) that is caused by SAH accumulation. In other words, shared SAM/SAH sensitivity of the involved methyltransferases constitutes an “Achilles heel” of the feedback mechanisms, which normally ensures stability of non-CG methylation patterns [10]. Consequently, manipulation of SAH levels constitutes an efficient way to alter non-CG methylation.

The reaction catalyzed by SAHH thermodynamically favors SAH production [57]. To avoid feedback accumulation of SAH, Hcy and Ado must be rapidly removed. Ado is removed by conversion to adenosine monophosphate (AMP), which is catalyzed by ATP-dependent Ado kinase (ADK) and constitutes the main salvage pathway in plants. Alternatively, Ado can be converted to inosine by Ado deaminase, but this pathway is not present in plants [ 66,67]. Reduced ADK activity in transgenic *Arabidopsis* lines is associated with dwarf phenotypes, SAH accumulation, and reduced pectin methylation of seed mucilage [66]. In animals, Hyc is removed either by conversion to cysteine (Cys) via the reverse transsulfurylation pathway or by being recycled to Met, either in a 5-methyltetrahydrofolate (5-CH_3_-THF)-dependent reaction that is catalyzed by methionine synthase (MS) or by folate-independent betaine-homocysteine *S*-methyltransferase homologs [68]. Due to the lack of reverse transsulfurylation, Hcy levels in plants are controlled by 5-CH_3_-THF-dependent MS activity as part of the methionine cycle and by homocysteine *S*-methyltransferase as part of the *S*-methylmethionine cycle [69].

MS from animals requires cobalamin (vitamin B12) as a cofactor for activation. As cobalamin is prone to non-catalytic oxidation, sustained MS activity requires regeneration of active cobalamin by MS reductase (encoded by *Mtrr*). Several mammalian studies have pointed toward an important role of cobalamin-dependent MS activity for epigenetic regulation [56,70,71]. A hypomorphic *Mtrr* mutation in mice has been associated with congenital malformations in wild-type grand progeny that were associated with altered DNA methylation and persisted for two additional generations [72]. In another study, genome-wide association mapping of sequence variation and CG methylation patterns in 90 mouse strains revealed a quantitative trait locus hotspot near *Mtrr.* Correlation with *Mtrr* expression further indicated that sequence variation at *Mtrr* affects CG methylation patterns [73].

In contrast, MS enzymes described in higher plants and fungi are cobalamin-independent. Nevertheless, the importance of proper Hcy recycling for the maintenance of DNA methylation was recently confirmed by a genetic screen for *Arabidopsis* silencing mutants that led to the identification of an *MS* hypomorph [11]. Accordingly, the identified *ms1* mutant showed decreased Met and increased Hcy content along with decreased SAM/SAH and epigenetic defects that were reminiscent of other mutants impaired in the methionine cycle. In agreement with this and other studies, MS1 seems to play a predominant role in Hcy recycling, as loss of function is lethal and not compensated by the other two paralogs in *Arabidopsis*, which are not required for DNA methylation or viability in general [11,74].

### 3.3 The methionine cycle, and hence transcriptional gene silencing, depend on folate metabolism

Folate metabolism provides one-carbon (C1) units for the synthesis of nucleotides, initiator tRNA, and pantothenate, as well as for the recycling of Hcy to Met [69]. The C1 units are carried by different intermediates of tetrahydrofolate (THF), ranging from reduced 5-CH_3_-THF to fully oxidized 10-formyltetrahydrofolate (10-CHO-THF). The corresponding C1-dependent pathways are compartmentalized in the cyto- and nucleoplasm, mitochondria, and plastids (Figure 1). The subcellular folate pools fulfill specific metabolic functions that are reflected in the THF composition [75,76]. For example, 5-CH_3_-THF is predominant in the cytoplasm, where it acts as a methyl donor for Hcy methylation as part of the methionine cycle [[77], [78], [79]]. Plant vacuoles also contain 5-CH_3_-THF, which probably serves solely as storage of 5-CH_3_-THF from the cytoplasm [79]. 5-CH_3_-THF is produced from 5,10-CH_2_-THF by methylenetetrahydrofolate reductase (MTHFR). In contrast to mammals, where this reaction is irreversible, there is evidence that MTHFR activity is reversible in plants [80]. Cytoplasmic 5,10-CH_2_-THF (the precursor of 5-CH_3_-THF) can be produced by either MTHFD1 or serine hydroxymethyltransferase (SHMT), which reversibly converts THF and serine (Ser) to 5,10-CH_2_-THF and glycine (Gly) [69].

Since the methionine cycle depends on the C1 supply from 5-CH_3_-THF, dietary and genetic folate deficiencies in humans and mice are frequently associated with elevated plasma Hcy levels [81] and can cause DNA hypomethylation [82]. In *Arabidopsis*, inhibition of folate biosynthesis by sulfonamide sulfamethazine (SMZ) as well as loss-of-function mutations in *FPGS1*, which encodes the plastidic folylpolyglutamate synthetase required for cellular folate homeostasis, lead to DNA and H3K9 hypomethylation and TE activation [9,83,84]. Rescue by supplementation of growth media with folinic acid (5-CHO-THF), which is readily taken up and converted to 5-CH_3_-THF, suggests that the epigenetic defects caused by SMZ or impaired FPGS1 function are linked to folate limitation [9,83,84]. The hypomorphic *mthfd1-1* mutant, which also has been identified by forward genetic screening for silencing defects, shows DNA and H3K9me2 methylation losses associated with Hcy and SAH accumulation. Although this is reminiscent of other mutants impaired in folate-dependent Hcy recycling, total leave tissue folate content, and in particular levels of 5-CH_3_-THF, were not altered in *mthfd1-1.* Moreover, supplementation with folinic acid did not rescue the *mthfd1-1* phenotype, which raised the open question of whether folate limitation causes Hcy accumulation in *mthfd1-1* or whether MTHFD1 might affect Hcy recycling by another mechanism [10].

The complexity of folate metabolism with its multiple interdependent subcellular pathways is reflected by the SHMT gene family in *Arabidopsis*, which contains seven members that have partially redundant compartment-specific functions. Among them, SHMT6 and SHMT7 are predicted to localize to the nucleus. Intriguingly, *shmt7/msa1 Arabidopsis* mutants, which have been identified during a genetic screen for leaf elemental composition and accumulated sulfur due to constitutive upregulation of sulfate assimilatory pathways, showed slightly reduced DNA methylation levels in roots that were associated with lower root tissue SAM levels [85]. Although SHMT7 did not show *in vitro* activity, the authors suggested that SHMT7 might be involved in nuclear SAM supply and that sulfur homeostasis is regulated through DNA methylation- and SAM-dependent mechanisms [85].

## 4 Redox-dependent chromatin modulation

### 4.1 Principles of redox signaling

Redox reactions are evolutionarily conserved signaling principles that occur in prokaryotes and eukaryotes. The most important redox molecules are reactive oxygen species (ROS) and nitric oxide (NO). ROS and NO are produced during normal energy metabolism (for example, respiration, photorespiration, and photosynthesis) and stress responses in plant cells. Cellular redox processes are extremely sensitive to environmental conditions. The nuclear compartment that orchestrates genetic programs of cell life is on the one hand particularly sensitive to the deleterious effects of oxidation and on the other hand contains fine-tuned redox-regulated signaling pathways. Experiments using isolated tobacco nuclei demonstrated that plant nuclei are an active source of ROS production, in particular H_2_O_2_ [86], and increasing evidence suggests an important role of redox signaling in the regulation of many nuclear proteins such as kinases, transcription factors, and chromatin modulators [87,88]. Modification of the cysteine redox state is the predominant redox signaling mechanism. Solvent-exposed thiols are prone to oxidation especially in a basic environment, which promotes thiol deprotonation and formation of a thiolate residue (R-S^−^). This nucleophilic residue is very sensitive to oxidation.

In the presence of ROS, such as H_2_O_2_ or superoxide, thiol groups are oxidized to sulfenic (R-SOH), sulfinic (R-SO_2_H), and sulfonic (R-SO_2_H) acids. Thiols can also be oxidized by oxidized glutathione (GSSG) resulting in S-glutathionylation (R-S-SG) [89,90]. Two closely located cysteine residues can also become oxidized to form a disulfide bridge (S–S). All of these modifications can alter the structure and/or activity of proteins in all of the cellular compartments, including chromatin modifiers in the nucleus.

NO is a ubiquitous signaling molecule with pleiotropic functions throughout the lifespan of plants. It is involved in several physiological processes, including growth and development, biotic and abiotic stress response, and iron homeostasis [[91], [92], [93], [94], [95], [96], [97], [98], [99], [100], [101]]. The multifunctional role of NO is based on its chemical properties, cellular environment, and compartmentation [102,103]. NO has been described as a cytoprotective as well as cytotoxic molecule depending on its local concentration, which is affected by its rate of synthesis, displacement, and removal [103,104]. The radical nature of NO promotes its interaction with different macro-molecules to transduce its bioactivity. Endogenously synthesized or exogenously applied NO can react with other radicals, including ROS, resulting in the formation of reactive nitrogen species (RNS), or with metals forming metal–nitrosyl complexes such as dinitrosyl–iron complexes (DNIC; formed by the interaction between NO, iron, and thiol-containing ligands such as glutathione (GSH), cysteine, or protein thiols) [105,106]. NO exerts its biological function through post-translational modifications (PTMs), including tyrosine nitration, metal nitrosylation, and S-nitrosation. These NO-mediated PTMs have profound effects on the function of target proteins by regulating their activities, subcellular localization, structure, or interaction with biomolecules, and consequently induce diverse physiological responses and/or signaling processes, including alteration of gene expression metabolic changes and phytohormone signaling [[107], [108], [109], [110], [111], [112], [113], [114]]. The most intensively studied signal transduction mechanism of NO is protein S-nitrosation. Proteome-wide studies identified putative S-nitrosated proteins involved in primary metabolism, defense response, and transcription regulation [[115], [116], [117], [118]]. More detailed studies demonstrated that NO directly regulates transcription by modifying transcription factors such as TGA1 [119], MYB30 [120], and SRG1 [121]. However, several lines of evidence also indicated that NO regulates gene expression via modification of chromatin accessibility [112,[122], [123], [124], [125]]. Consequently, the redox status of plant cells has the potential to control chromatin modifications and epigenetic reprogramming of gene expression [[126], [127], [128], [129], [130], [131]]. The following sections discuss the regulatory function of redox molecules on covalent modifications of core histones, DNA methylation, and metaboloepigenetic effects.

### 4.2 Redox regulation of histone acetylation

Redox regulation of epigenetic processes has mostly been addressed in mammals [[122], [123], [124], [125], [126], [127]]. Multiple studies have provided evidence that histone deacetylation activities are sensitive to redox modification resulting in altered chromatin conformation and transcriptional changes [123,132,133]. Different enzymes responsible for regulating H3K9ac and H3K14ac seem to be targets for redox regulation. For example, a ROS/thioredoxin (TRX)-dependent redox switch of key Cys residues regulates nuclear trafficking of class II HDAC4 [133] and S-nitrosation of HDAC2 at conserved cysteine in neurons affects histone modification and gene expression [123]. However, this topic has only recently been explored in plants [28,131,134,135]. Since several histone acetylation mechanisms are largely conserved in eukaryotes, it is likely that distinct plant HDACs are also redox-regulated. Within the 18 identified HDACs in plants [17], members of the class I RPD3-like HDAC (HDAC6, HDAC9, and HDAC19) might be sensitive to oxidation [112,131,136]. Human and plant HDACs share a domain that contains six highly conserved cysteine residues. Interestingly, the cysteine residues that are targeted by NO in human HDAC2 (Cys262 and Cys274) are located within this region, and structural modeling of the plant HDAC domain based on the available crystal structure of human HDAC2 revealed a strikingly similar 3D-fold of these proteins [131]. The two conserved potential NO-sensitive cysteine residues of plant HDACs are located close to the substrate binding site at the same positions as in human HDAC2. This makes plant HDAC6, HDCA9, and HDAC19 promising candidates for NO-affected/regulated nuclear HDAC isoform(s).

Chemical treatment with an NO donor, S-nitrosoglutathione (GSNO), and subsequent demonstration of specific changes in H3K9/14 acetylation patterns in *Arabidopsis* seedlings by ChIP-seq has been conducted to further investigate the effect on histone acetylation [112]. Most of the observed alterations were found within genes involved in the response to biotic and abiotic stresses. The majority of NO-regulated H3K9/14ac sites were similarly affected by trichostatin A treatment (a strong inhibitor of RPD3/HDA1-like HDACs), indicating that these changes might be induced by NO-dependent inhibition of HDAC activity. Supporting this, a slight but significant increase in total H3ac was observed upon treatment of *Arabidopsis* suspension cells with GSNO. Moreover, salicylic acid induced a rapid and strong endogenous NO production in plant cells, which correlated with a decrease in HDAC activity, and scavenging of NO or treatment with a reducing agent rescued HDAC activity, indicating that the inhibition was mediated by S-nitrosation [112]. In conclusion, NO-dependent regulation of plant HDACs can be considered a key mechanism in the regulation of histone acetylation and gene expression. NO-dependent regulation of plant HDACs might play a role during the plant’s stress response to enhance transcription of stress-related genes by inducing histone acetylation at the corresponding loci.

### 4.3 Redox-dependent modulation of histone and DNA methylation

In mammals, NO has been shown to directly and indirectly affect DNA and histone methylation (reviewed in Refs. [[126], [127], [128], [129], [130]]). NO induces transcriptional changes of DNA and histone methyltransferases (HMTs) and demethylases. For example, the expression levels of H3K9me HMTs are differentially controlled by NO in human cells. Upon treatment with NO, the expression levels of SETDB2 and SUV39H2 (both tri-methylate H3K9) increased, while the levels of G9a (di-methylate H3K9) decreased [130,137]. Application of RRX-001, a compound generating reactive nitrogen and oxygen species, decreased the expression of DNA methyltransferases (DNMTs) in mammalian cells and diminished global DNA methylation levels [138]. Conversely, exposure of mammalian cells to NO donor SNAP did not change the expression of DNMTs [139].

NO-induced alteration of enzymatic activities of chromatin-modifying enzymes was also observed. For instance, the activity of DNMTs increased when NO was applied directly to a nuclear protein extract [139]. Similarly, endogenously produced NO upon *Helicobacter pylori* treatment was associated with increased DNMT activity and an increase in DNA methylation [140]. Multiple studies have indicated that HMTs are also prone to redox modification [[141], [142], [143]]. Moreover, it is now well established that NO inhibits mononuclear non-heme iron dioxygenases such as JHDM (histone demethylase) and TET (DNA-demethylase) enzymes by forming a nitrosyl–iron complex with their catalytic non-heme iron [137,144]. For instance, the NO-mediated inhibition of recombinant human lysine demethylase 3A (KDM3A) by forming a nitrosyl–iron complex in the catalytic pocket prevents the binding of the co-substrate O_2_ required for demethylase activity [137]. Accordantly, exposing cells to NO resulted in a significant increase in H3K9me2, the preferred substrate for KDM3A [137]. Similarly, the NO donor DETA-NO caused global increases in the levels of H3K9me2, H3K9me3, and H3K36me3 to the same extent as the dioxygenase inhibitor dimethyloxalylglycine (α-KG analog) in macrophages. Additionally, NO affects the activities of JmjC domain-containing histone demethylase (JHDMs) by sequestration of chelatable iron, which is considered the major source of iron for JHMDs via the formation of dinitrosyl–iron complexes [137].

NO can also lead to enzymatic degradation, as shown for the H3K9 tri-methylating HMT SUV39H1. Neurotrophin-induced NO production resulted in S-nitrosation of GAPDH enabling its nuclear translocation in complex with seven in absentia homolog 1 (SIAH1) protein, a known ubiquitin E3 ligase. The GAPDH-SIAH1 complex associated with SUVH39H1 mediated its degradation, which in turn resulted in decreased H3K9me3 levels [145]. Taken together, redox events have emerged as important mechanisms to regulate chromatin modifiers on different levels (transcription, activity, and degradation of chromatin modifiers), resulting in altered histone methylation patterns and DNA methylation in mammalians.

In plants, different studies have analyzed transcriptome changes upon exogenous NO application or in plants with impaired NO homeostasis. Interestingly, many of the differentially expressed genes had annotations linked to DNA or histone methylation (summarized in Table 1). For instance, genes involved in *de novo* and maintenance DNA methylation as well as in active demethylation were differentially expressed upon treatment of *Arabidopsis* leaves with NO donor CysNO [146]. Intriguingly, *de novo* methylation and maintenance pathways showed opposite effects, as various components and positive regulators of the RdDM pathway, including SUVH9 and lysine-specific demethylase 1-like 1 (LDL1), were downregulated, whereas CMT2 and agenet domain-containing P1, which links H3K9me2 to DNA methylation in heterochromatin [147], were upregulated. Additionally, CysNO treatment resulted in downregulation of ROS1, the major DNA demethylase in *Arabidopsis* [146]. It is possible that NO exerts different regulatory effects during establishment and maintenance of DNA methylation or on euchromatic vs pericentromeric heterochromatin, as the former is targeted by RdDM, whereas the latter is methylated by CMT2.

**Table 1:** **NO-induced changes in expression of genes related to histone and DNA methylation**. Previously reported transcriptomic analyses were screened for differentially expressed genes involved in histone and DNA methylation pathways.

Treatment (citation)	Up-/down-regulated	Gene(s)	Function(s)/pathway(s)
Infiltration of 4-week-old leaves with 1 mM CysNO [145]	Up	STABILIZED1, RDM16, HSP90-1	RdDM
		CMT2	CHG/CHH methylation
		AGDP1	AGDP1 linking DNA and H3K9me2
		SUVR3/SDG20 JMJ13, JMJ21, JMJ26, JMJ29	Histone methylation Histone demethylation
	Down	ROS1, DML2, IDM3	DNA demethylation
		NRPD2/NRPE2, NRPE5, AGO4, DMS3/IDN1, KTF1, IDP1, IDP2, SUVH9, LDL1, RRP6L1	RdDM
		DDM1	DNA methylation
		SWN/SDG10, SHR3/SDG4, ATX5/SDG29, SUVH9/SDG22, SUVR2/SDG18, PRMT4A JMJ27, LDL1	Histone methylation Histone demethylation
		DML2	DNA demethylation
Microarray analysis WT vs *noa1-2*, decreased NO level [152]	Up	NRPD4/NRPE4, NRPE5, HSP90-1	RdDM
		MET1, VIM1	CG methylation
		ATXR7/SDG2, PRMT1a, PRMT1b, PRMT3, PRMT10, PRMT5, JMJ22	Histone methylation
	Down	CMT2	CHH methylation
		ASHH3/SDG7, SUVR2/SDG18 JMJ18	Histone methylation Histone demethylation
		NBP35	CIA pathway
Microarray analysis WT vs *nia1nia2*, decreased NO level [152]	Up	RDM1	RdDM
		PRMT1b, PRMT3, PRMT4B	Histone methylation
		ROS1, DML2	DNA demethylation
	Down	DCL3, DRD1, IDN1, LDL1, BP26, RRP6L1, kRDM16	RdDM
		MET1, CMT2	DNA methylation
		ASHH3/SDG7, ATXR6/SDG34, SUVH1/SDG32 JMJ11, JMJ18, JMJ28	Histone methylation Histone demethylation
		APE1L	DNA demethylation
Microarray analysis WT vs *nia1nia2noa1-2*, decreased NO level [152]	Up	RDM1	RdDM
		PRMT1b, PRMT3, PRMT4B, RMT10, PRMT5 NAR1, DRE2, NBP35, CIA1	Histone methylation CIA pathway
		ROS1, MBD7	DNA demethylation
	Down	AGO4	RdDM
		CMT2	CHH methylation
		ATX5/SDG29, ATXR6/SDG34, UVH6/SDG23, SUVR2/SDG18 JMJ18	Histone methylation Histone demethylation

Further, CysNO altered the expression of genes encoding JHMDs [146]. Because JHMDs require iron for their activity, genes encoding nicotianamine synthase (that converts SAM into nicotianamine) isoforms are differentially regulated upon NO donor treatment [146,148] and in mutants with impaired NO homeostasis [[148], [149], [150], [151], [152]]. Nicotianamines are high-affinity metal chelator molecules that play a key role in Fe homeostasis and transport in plants. Transcriptomic analysis of NO-deficient *noa1-2*, *nia1nia2*, and *nia1nia2noa1-2* mutants also revealed that genes encoding for enzymes involved in epigenetic methylation processes are differentially expressed [153]. For instance, CMT2, responsible for CHH methylation at pericentromeric heterochromatin, were downregulated in each mutant [153]. Additionally, MET1 maintaining CG methylation was upregulated in *noa1-2* but downregulated in *nia1nia2*. Further, genes encoding for enzymes involved in active DNA demethylation such as ROS1 and DML2 were differently expressed in these NO-deficient mutants. Many genes encoding essential components of the CIA pathway were also upregulated in *nia1nia2noa1-2*. The CIA pathway is required for maturation of Fe-S cluster proteins such as ROS1 [154]. Interestingly, the expression of several protein arginine methyltransferases (PRMTs) was upregulated in NO-deficient plants. For instance, PRMT1b, which methylates H4R3, was upregulated in all three NO-deficient mutants [155]. Another example is PRMT5, which catalyzes symmetric di-methylation of H4R3 *in vitro* and is essential for proper pre-mRNA splicing [156]. PRMT5 was upregulated in *noa1-2* and *nia1nia2noa1-2* 44 and positively regulated by S-nitrosation during stress responses [157]. Regarding the late flowering phenotype of these NO-deficient mutants, JMJ18 was downregulated in each mutant. JMJ18 is a H3K4 demethylase that controls flowering time. Enhanced endogenous levels of NO or GSNO, due to overexpression of rat neuronal NOS (nNOS) or knock-out of GSNOR, respectively, resulted in downregulation of JMJ30 [149,150]. JMJ30 demethylates H3K36me2/3, regulates period length in the circadian clock [158], and is involved in the control of flowering time. In sum, the expression of DNA and histone methylation-modifying enzymes is differentially controlled by NO. This implies an indirect effect of NO on epigenetic mechanisms in plants. Clearly, these observations are still very preliminary, as expression patterns from different treatments are not consistent and partially contradictory, and verification of the resulting DNA and histone methylation patterns remains lacking. The complexity was further illustrated by a study in rice (*Oryza sativa* L. spp. Japonica), in which high concentrations of the NO donor sodium nitroprusside induced global DNA hypomethylation (mainly in CHG sites) and led to altered expression of chromatin remodeling enzymes [159].

Although proteome-wide studies identified several proteins involved in DNA and histone methylation as candidates for S-nitrosation, functional analyses of these S-nitrosated proteins are rare. Only S-nitrosation of PRMT5 has been described in detail [157]. Briefly, S-nitrosation of PRMT5 in *Arabidopsis* promotes its methyltransferase activity, which enables methylation-dependent pre-RNA splicing associated with salt stress tolerance [157]. Further, Argonaute 4 (AGO4), a component of the canonical RdDM pathway, was identified as putatively S-nitrosated in a proteome-wide approach [116]. Based on studies of human JHDM [137], Fe(II)-dependent plant JHMDs might be targets for metal nitrosation by the formation of a nitrosyl–iron complex with the non-heme Fe(II) in their catalytic pocket. Hence, NO-induced changes of histone methylation by the direct inhibition of the catalytic activity of plant JHMDs are suggested. In this regard, because iron-sulfur clusters of proteins are targeted by NO resulting in the disruption of the cofactor, the iron-sulfur containing ROS1/DME DNA demethylases might be affected by NO in plants.

### 4.4 NO affects metaboloepigenetic processes interacting with histone and DNA methylation

Several proteome-wide studies of plants revealed key enzymes of the methionine cycle as targets for S-nitrosation, namely MS, SAMS, and SAHH [115,[160], [161], [162], [163], [164], [165]], and for tyrosine nitration, MS and SAHH [[166], [167], [168]], suggesting that NO affects the methionine cycle. In mammals, NO inhibits cobalamin-dependent MS due to its reaction with the cofactor cobalamin [[169], [170], [171]]. In plants, cobalamin-independent MS isoforms were identified as targets for S-nitrosation [115,165] and nitration [166]. In *Arabidopsis*, SAMS isoforms are differentially inhibited by protein S-nitrosation [164]. While SAMS1 is reversibly inhibited by GSNO, SAMS2 and SAMS3 are not affected. It was demonstrated that S-nitrosation of the Cys114 of SAMS1, which is located next to the catalytic center as part of the active site loop, is responsible for inhibition [164]. A similar differential regulation of SAMS activity was observed in mammals. Here, two genes encode different SAMS isoforms. Only the isoform SAMS1A, but not SAMS2A, was reversibly inactivated by NO [172]. Additionally, tyrosine nitration has been observed in SAHH of sunflowers, causing decreased SAHH activity [168]. Further, site-specific S-nitrosation of SAHH1 protein upon cold stress was reported, but the physiological consequence of cold stress-induced S-nitrosation of SAHH1 has not yet been investigated [117,173]. Of note, O-acetylserine(thiol)-lyase is a target for S-nitrosation [115] and is inhibited by the nitration of the tyrosine residue [174,175]. This enzyme is essential for the biosynthesis of cysteine and methionine [176], which is converted into SAM in the methylation cycle. Taken together, NO may play a regulatory role in different enzyme activities for the synthesis of sulfur-containing amino acids and the methylation cycle providing the methyl-group donor SAM for transmethylation reactions. In addition to the identification of proteins that are targets for NO-induced PTMs, the effect of NO on metabolic reprogramming using untargeted and targeted metabolomics together with transcriptomic profiling is an emerging field. In this regard, transcriptomic profiling upon NO donor treatment and in mutants with altered NO homeostasis revealed that genes involved in methyl donor supplies are regulated by NO. For instance, treatment of *Arabidopsis* cell suspension with 0.5 mM NOR3 resulted in downregulation of MS1, SAMS3, and SAMS4. In contrast, exposure of 4- to 5-week-old *Arabidopsis* plants to gaseous NO resulted in an induction of SAMS4 [177]. Moreover, CysNO treatment induced the expression of SAMS2, SAMS3, SAMS4, SAHH1, and SAHH2. In addition, genes involved in folate, cysteine, methionine, and GSH biosynthesis and methylation cycles were differentially regulated upon CysNO treatment [146]. Transcriptomic profiling of NO-deficient mutants *noa1-2*, *nia1nia2*, and *nia1nia2noa1-2* revealed that genes coding for enzymes involved in the methylation cycle, folate, cysteine, methionine, and GSH biosynthesis are differentially regulated [149]. An untargeted metabolomic analysis recently revealed that NO affects glutathione, methionine, and carbohydrate metabolism in elm seeds [178]. In particular, SNP and GSNO modulate the methylation cycle at the transcriptional level by elevating SAMS transcript levels and increasing the levels of methionine and SAM [178]. In sum, these data demonstrate that NO affects the methyl donor supply by altering the transcription of genes coding for enzymes involved in SAM biosynthesis, changing enzyme activities and altering metabolic levels.

Transcriptomic profiling also revealed numerous genes involved in the TCA cycle that are differentially expressed upon CysNO treatment or in NO-deficient mutants (*noa1-2*, *nia1nia2*, and *nia1nia2noa1-2*) [146,153]. For instance, aconitase 2 and 3 (ACO2 and ACO3) were upregulated in *nia1nia2* and *nia1nia2noa1-2*. Further, it was demonstrated that NO inhibits aconitase by forming a metal–nitrosyl complex with an iron-sulfur cluster [179,180]. In addition, ACO was found as a target for S-nitrosation [160,181]. It is known that under stress conditions, NO regulates the cellular redox state, which might influence the availability of FAD and thus the activity of histone demethylase LSD1 [182]. Further, an untargeted metabolic analysis revealed that plants exposed to NO for six hours undergo transient metabolic reprogramming, including increased succinate (inhibit JHDMs) and decreased α-KG (substrate of JHDMs) levels [113]. Both NO-deficient mutants *nia1nia2* and *nia1nia2noa1-2* displayed an impaired TCA cycle. The *nia1nia2noa1-2* mutant plants demonstrated a significantly increased level of succinate and a decreased level of fumarate (inhibit JHDMs), whereas α-KG was not altered compared to wild-type [183]. In contrast, the succinate and fumarate levels were decreased in the *nia1nia2* mutants [184]. Hence, it is suggested that NO may regulate histone methylation by effecting these metabolites. Taken together, NO is supposed to be an epigenetic regulator of DNA and histone methylation in plants based on *S*-nitrosoproteomic studies, transcriptomic profiling, and metabolic analysis using exogenous NO donor treatments and mutants with impaired NO homeostasis.

## 5 Interactions of redox signaling, metabolism, and chromatin modification in response to environmental stress

The ability to adjust cellular programs to environmental conditions seems particularly pronounced in plants [185]. Plants are generally sessile and therefore cannot evade biotic and abiotic stresses. Perennial plants are challenged by seasonal fluctuations and long-lived tree species and even face climate-change phenomena during their lifetimes. Epigenetic mechanisms have been postulated to confer phenotypic plasticity and stress memory in plants. Although environmental responses are often temporary, life-threatening events, such as heat or pathogen attacks, can elicit systemic responses and sustained morphological and physiological provisions for recurring stress that are known as acclimation or priming [186]. Enhanced stress tolerance may even be inherited, but in most examples, transgenerational stress memory is erased within one stress-free generation [34,187,188]. Nevertheless, the notion that epigenetic mechanisms are at the core of stress memory has been substantiated by studies showing that specific histone modifications, in particular histone H3K4 di- and tri-methylation, and chromatin remodeling factors are involved in the priming of stress response genes [189,190]. Importantly, chromatin modifications can thereby act as a memory and lead to faster or stronger gene responses during subsequent stress [187,188,191].

To date, numerous methylome studies of *Arabidoposis*, rice, and other plant species have shown altered DNA methylation patterns upon different environmental challenges, including phosphate starvation, hyperosmotic stress, chemical elicitors, and pathogen treatment [188,[192], [193], [194]]. For example, the bacterial pathogen *Pseudomonas syringae pv. tomato* (*Pst*) as well as hyperosmotic stress lead to sizeable changes in somatic DNA methylation patterns in challenged plants and their direct progeny [188,192,193]. Moreover, increased resistance to bacterial pathogens as well as hyperosmotic stress in the progeny is associated with specific DNA methylation patterns and lost in DNA methylation and DNA glycosylase mutants, indicating that DNA methylation changes at RdDM target sites might cause stress memory and control the inducibility of specific response genes [188,195].

**Figure 2:**
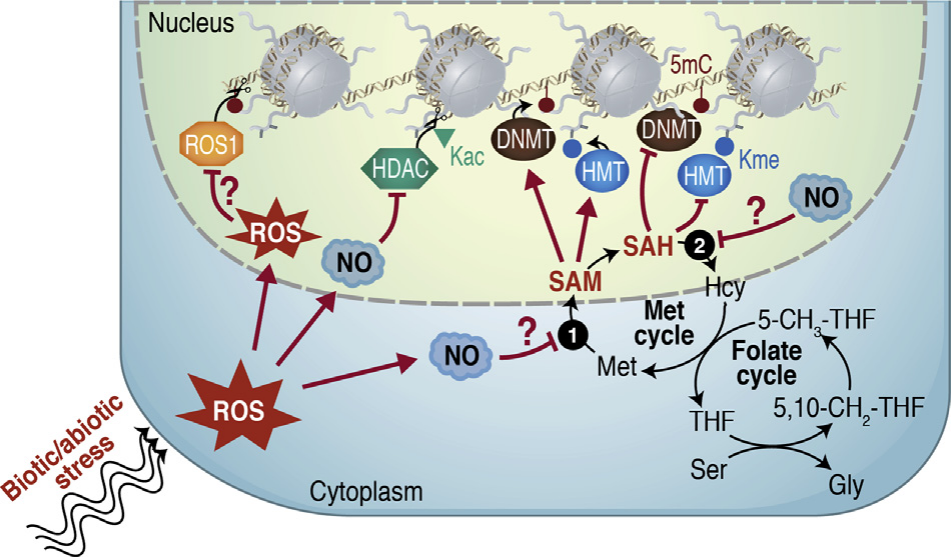
**Model of stress interactions of metabolism and chromatin modification.** Environmental stress leads to increased production of reactive oxygen species (ROS) and NO. ROS may lead to inactivation of DNA glycosylases involved in DNA demethylation (for example, ROS1). NO acts as an HDAC inhibitor and may also affect the activity of enzymes in the methionine (Met) cycle, for example, SAMS4 (1) and SAHH1 (2), leading to changes in SAM and SAH levels. This can lead to changes in histone lysine methylation (Kme) and cytosine methylation (5mC), as DNA methyltransferases (DNMTs) and histone methyltransferases (HMTs) require SAM as methyl donor and are inhibited by SAH. The Met cycle depends on C1 supply from the folate cycle.

Although expression changes of RdDM genes and DNA glycosylases, as observed upon *Pst* treatment, might account for some of these DNA methylation dynamics [191], the involved mechanisms are not well understood. In contrast, it is well known that biotic and abiotic stress leads to profound metabolic responses that are commonly characterized by an immediate burst in ROS production, often followed by redox-mediated signaling mechanisms [6,196,197]. Based on the outlined reports on thiol-based redox-regulated nuclear proteins, there is increasing support for the concept that redox-mediated signaling affects epigenetic regulation by changes in the activity of histone and DNA-modifying enzymes (Figure 2). In this context, it is noteworthy that oxidative stress has also been implicated with human pathologies that are connected to altered DNA methylation, particularly cancer [198]. ROS/NO may directly affect the activity of chromatin modifiers or regulate the supply of methyl group donors and methylation inhibitors via redox-dependent protein modifications (Figure 2). Production of primary and secondary plant metabolites that depend on SAM and act as protectants, detoxification agents, or hormones, such as betaine glycine, spermidine, and ethylene, can also lead to increased C1 demands upon stress and thereby competition for potentially limited folate pools [69,199]. Due to the described sensitivity of DNA and histone methyltransferases as well as DNA glycosylases to redox/NO signaling, and C1 homeostasis, it is therefore possible that metabolic shifts can cause enhanced or reduced chromatin methylation in response to environmental cues. In this context, folate-mediated changes in DNA methylation were recently linked to altered pathogen resistance toward *Pst* [74]. Using a chemical genetic approach, the authors showed that antifolates, which are known to block the methionine cycle and thereby interfere with DNA methylation in plants, enhance resistance against *Pst*, whereas overexpression of *MS1* led to DNA hypermethylation, particularly in the non-CG context, and attenuated resistance [74].

## 6 Conclusions and outlook

Several proteins involved in primary or secondary metabolism also participate in chromatin modulation and gene regulation by providing cofactors or substrates for epigenetic modifiers, highlighting the strong interconnection of metabolism and epigenetics. However, the understanding of the detailed function of metabolites in epigenetic regulation during stress responses remains in its infancy. One of the challenges in the field is the dissection of the many pleiotropic ramifications of metabolic regulation to distinguish between direct and indirect effects. Experiments using NO donor treatments have shown some interesting expression changes in genes encoding known chromatin-modifying proteins, some of which are known to be redox sensitive in animals. However, complementary analyses of chromatin modification patterns are missing in most cases, and even if present, the causalities are difficult to determine, as chromatin modification and transcriptional dynamics often go hand-in-hand. One approach that has been successfully used to describe redox regulation mechanisms in other biological contexts is based on the identification of SNO and other redox-dependent PTM targets using proteomics. Following their identification, *in vitro* and *in vivo* functional characterization of their redox-dependent activities, substrates, and/or interaction partners can provide valuable new insights into the signaling of environmental cues at the chromatin level.

The interactions between epigenetics and metabolism are not unidirectional and involve complex regulation, e.g. during environmental response or development. How and under which circumstances distinct metabolic changes impact chromatin structure or vice versa still needs to be investigated in more detail. For instance, increased acetyl-CoA is accompanied by an increase in H3K27ac, which can result in altered α-ketoglutarate levels [26]. α-Ketoglutarate in turn is an important metabolic intermediate acting as cofactor for several chromatin-modifying enzymes [27]. This demonstrates how primary metabolism can specifically affect chromatin structure, on the one side, and how these chromatin changes affect specific metabolic pathways, on the other side [26]. However, such mutual relationships have to be addressed in more detail in the future, e.g. by time-course studies in combination with inducible inhibition/activation of genes/proteins involved in the production of redox molecules, cofactors, and other intermediates.

It is presently well established in animals and plants that the levels of metabolic intermediates often globally correlate with the levels of epigenetic modifications for which they serve as cofactors. However, the biological implications, such as connections between diurnal metabolic changes and the circadian clock, are largely unknown in plants. On the one hand, a better picture is needed of the dynamics, tissue specificities, and subcellular distributions of the involved metabolites, including folate species and thiols, which also requires further development of the quantification methods. On the other hand, to what extent and how cofactors levels are involved in the regulation of specific chromatin modification pathways or certain genomic regions must be addressed. Regarding histone methylation, different studies of *Arabidopsis* indicate that DNA methylation and H3K9me2 are particularly sensitive to changes in SAM and/or SAH levels [9,200], and the likely connection to the feedback mechanism between these two chromatin marks has been discussed. In comparison, reduced SAM and SAH levels due to Met restriction in human cells mostly affected H3K4me3 [201]. It is known that histone methyltransferases differ in their binding affinities toward SAM and SAH [202], which also might lead to different sensitivities of the corresponding methylation marks toward metabolic fluctuations. Furthermore, varying global and regional dynamics and turnover rates of chromatin marks and nucleosomes can also affect cofactor dependencies. Another mechanism providing specificity for different pathways and certain genomic regions constitutes metabolic channeling through enzyme complexes. In mammals and yeast, recent studies suggested that nuclear components of the methionine cycle are involved in epigenetic regulation by directly interacting with histone methyltransferases. For example, it has been shown that the catalytic subunit of mammalian SAM synthetase, MATIIα, is recruited to loci involved in oxidative stress and immune response, where it interacts with different chromatin-associated proteins, including the histone methyltransferases SETDB1 and SUV39H1, presumably to provide SAM for H3K9me3 [203]. Remarkably, MATIIα activity is not required for H3K4me3 at these loci. In yeast, it has been shown that SAM synthetase associates with a chromatin-modifying complex that includes metabolic enzymes and thereby may coordinate metabolic states with epigenetic regulation [204]. Such complexes have not yet been described in plants; however, plant nuclei show defined folate metabolic activities, and many proteins involved in folate-mediated C1 metabolism are localized in the cytosol and the nucleus [78,205]. For some of these proteins, nuclear methylation functions have been proposed [206,207], but their roles in chromatin dynamics and epigenetic regulation remain elusive.

It is noteworthy that several pathways involving methionine cycle intermediates are not conserved between plants and animals, which may be why many genes associated with elevated plasma Hcy in humans do not have homologs in plants (for example, *GNMT*, *CBS*, and *ALDH1L1*) [208]. In addition, plants might have evolved alternative ways to control Hcy and SAH levels. Their steady-state-levels seem to be tightly controlled [209] and affected by environmental factors, e.g. nutrient availability [210]. The mechanisms controlling Hcy and SAH levels have not been thoroughly investigated in plants, but due to the sensitivity of DNA and histone methylation to changes in SAM/SAH levels, they might eventually include as-yet-unknown factors involved in chromatin methylation dynamics.

Another challenge is the separation of genetic and epigenetic effects. To this end, the use of epigenetic recombinant inbred lines (epiRILs) in *Arabidopsis* has been seminal, leading to the identification of DNA methylation-dependent quantitative traits [211]. Further analyses along those lines with a particular focus on metabolic traits may lead to the identification of new mechanisms involved in epigenetic regulation. Finally, the identified mechanisms and the natural environment have to be analyzed in a developmental context to complement the picture of ROS/NO signaling, metabolism, and epigenetic functions in plants. Large-scale analyses of natural genetic and epigenetic variations in *Arabidopsis* linked changes in DNA methylation to environmental adaptations are key [[212], [213], [214]]. The tracking of epigenetic variations over multiple generations under environmental simulation of projected climate parameters, including CO_2_ levels, temperature, and soil water content, will be instrumental to define long-term environmental effects on epigenetic regulation and the possible impact of global warming on chromatin modification patterns. As stated initially, our overarching interested in the interactions between metabolism and chromatin lies in the environmental impact on epigenetics, which is relevant from the health, agricultural, and ecological perspective, alike. Elucidating these interactions will require interdisciplinary approaches and can greatly benefit from the use of different model systems, including plants, as hopefully conveyed by this review.

## Conflict of interest

The authors declare that there are no conflicts of interest.
